# An unusual case of symptomatic deep vein thrombosis and pulmonary embolism after arthroscopic meniscus surgery

**DOI:** 10.1186/s12891-017-1919-0

**Published:** 2018-01-17

**Authors:** Chao-Hua Fang, Hua Liu, Jun-Hui Zhang, Shi-Gui Yan

**Affiliations:** 1 Department of Orthopaedic Surgery, Second Affiliated Hospital, School of Medicine, Zhejiang University, No.88 Jiefang Road, Hangzhou, 310009 People’s Republic of China; 2 Department of Joint Surgery, the 6th Hospital of Ningbo, Ningbo, 315000 Zhejiang People’s Republic of China

**Keywords:** Pulmonary embolism, Deep vein thrombosis, Arthroscopy, Meniscectomy, Thromboprophylaxis

## Abstract

**Background:**

Although thrombosis complication is rare after arthroscopic meniscus surgery, deep vein thrombosis and pulmonary embolism can be fatal. The associated risk factors and whether anticoagulant prevention after arthroscopic knee surgery is necessary have not reach consensus. Here we present a case of deep vein thrombosis and pulmonary embolism after a common arthroscopic meniscectomy.

**Case presentation:**

The patient had no risk factors except ipsilateral leg varicose veins. She present swell at knee and calf from postoperative 3 weeks, and developed dyspnea, palpitation, and nausea on 33th day, pulmonary embolism was confirmed with CT angiography at emergency department. After thrombolysis and anticoagulation therapy were administered, the patient improved well and discharged. And the intravenous ultrasound confirmed thrombosis of popliteal vein and small saphenous vein. Who don’t have common risk factors for venous thromboembolism.

**Conclusions:**

Despite the low incidence of thromboembolic complications after simple arthroscopy surgery, its life-threatening and devastating property make clinicians rethink the necessity of thromboprophylaxis and importance of preoperative relative risk factors screening.

## Background

Although reported occasionally, deep vein thrombosis (DVT) and pulmonary embolism (PE) secondary to knee arthroscopy is rare. To the best of our knowledge, few report on this complication after arthroscopic meniscus surgery exists in literatures [1–4]. The arthroscopic meniscus surgery is characterized by low-difficulty, minimally invasive and fast recovery and make surgeon suppose its low risks of thromboembolic complications. Actually the incidence of symptomatic postoperative venous thromboembolism (VTE) is low as well, although post-arthroscopy VTE diagnosed by venography is relatively high [5]. In addition, this complication can be regard as most potentially life-threatening and devastating and need to be taken seriously. Here we present a case of DVT subsequent PE confirmed by ultrasonography and CT angiography (CTA) after arthroscopic meniscectomy.

## Case presentation

Arthroscopic isolated partial meniscectomy was performed in a 53-year-old female with MRI confirmed degenerative tear in the posterior horn of the medial meniscus. She manifested pain at medial side of the left knee joint, but cannot be alleviated after two weeks medication of oral NASIDS. The operation was done smoothly under subarachnoid anesthesia without using a tourniquet, and cost only 20 min. Degenerative tear in the posterior 1/3 medial meniscus but no obvious chondral lesion were found intraoperatively. The patient was slim, with the BMI of 23.7, and had no malignancy, VTEs and vascular diseases history but ipsilateral leg varicose veins, no use of anticoagulant and hormone. Also no one in her family had history regarding VTE events. As a routine procedure of thromboprophylaxis, the patient was encouraged to perform ankle pump and continuous passive motion (CPM) exercises after surgery but no use of elastic stockings, and 3200 IU low molecular weight heparin calcium(LMWHC) was injected subcutaneously at the morning of following day. Then she was allowed walking and discharged, flexion and extension of knee joint without weight bearing and straight leg-raising exercises were encouraged but long distance walking was discouraged at home.

**Fig. 1:**
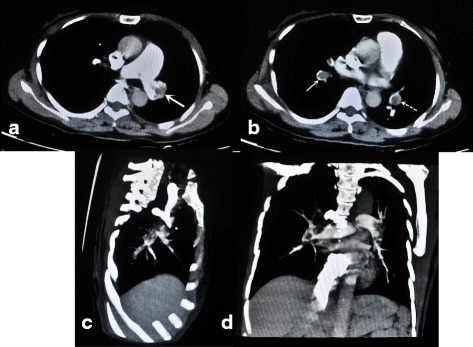
**a** show the thrombosis at left main pulmonary artery in chest CT angiography. **b** show the obstruction in both pulmonary arteries (solid arrow:right side, dotted arrow:left side). Extensive obstruction in both pulmonary arteries were showed in lateral view(**c**) and frontal view (**d**) of chest CT angiography

**Fig. 2:**
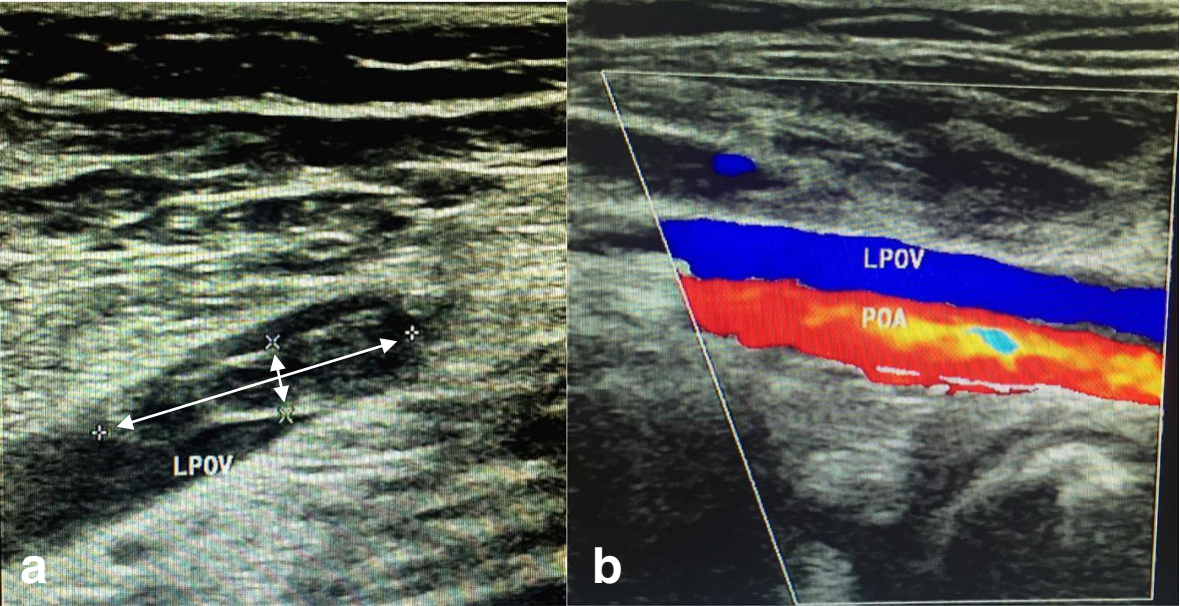
**a** show the thrombosis in the left popliteal vein(LPOV), the size is about 21 mm × 5 mm as indicate by asterisk. **b** show the blood flow in left popliteal artery(LPOA) and partial recanalization of left popliteal vein

Three weeks after surgery, she found swell at the knee joint and it extend to calf and ankle sequentially, but had not attached importance to it. On the 33th postoperative day, dyspnea, palpitation, and nausea were felt abruptly after stand up, and these symptoms had not alleviated after a rest. Soon she presented with severe dyspnea, tachycardia, and hypotension at ambulance and was transfer to emergency department. Pitting edema in the calf was found. An arterial blood gases analysis showed decreased PO_2_ (72 mmHg, normal 80–100 mmHg) and PCO_2_ (31 mmHg, normal 35–45 mmHg). Coagulation function showed elevated D-dimmer (2494 ng/ml, normal 0–243 ng/ml) and FDP (28.88μg/ml, normal 0–5 μg/ml).Electrocardiography revealed sinus tachycardia and a right ventricular strain pattern, and chest CTA showed obstruction in both pulmonary arteries (Fig. 1). So she was diagnosed pulmonary embolism(PE) and was admitted into ICU, Urokinase (0.2 million units ivgtt in10 minutes, followed by 1.2 million units ivgtt for 10 h) thrombolysis was administered, enoxaparin(60 mg ih q12h) and warfarin(3.125 mg po qn) were prescribed subsequently for anticoagulation. One week later, her condition was stable and transfer to normal ward, the edema at calf also alleviated obviously. The intravenous ultrasound confirmed thrombosis of popliteal vein and small saphenous vein (Fig. 2). The reexamined chest CTA revealed pulmonary arteries obstruction had dissolved. After two weeks anticoagulation of warfarin(3.125 mg po qn), the thrombosis of popliteal vein vanished and the patient discharged. Warfarin was prescribed, and regular follow up was conducted to monitor the coagulation function and residual small saphenous vein thrombosis in outpatient department.

## Discussion

It is extensively accepted that thromboprophylaxis is necessary and perioperative thrombosis prevention treatments are recommended in many guidelines for major orthopaedic surgeries, such as joint replacement and trauma-related procedures [6, 7]. But non consensus have been reached about thromboprophylaxis after arthroscopic surgery. Although several cases of thrombosis complications have been reported, it is deemed risk of a thromboembolic complication is low in arthroscopic knee surgery, prophylaxis against thromboembolism has been thought to be unnecessary [8–10]. The incidence of post-arthroscopy VTE diagnosed by venography reach as high as 14.9% according to a retrospective study of 537 consecutive patients, but distal and asymptomatic clots are generally considered to be clinically insignificant [11]. And the incidence of symptomatic VTE after arthroscopic meniscectomy decrease to approximately 0.34% [12]. Comparing with ligament reconstructions, other arthroscopic surgery including meniscal surgery, diagnostic arthroscopy, or chondroplasty possess a lower risk of thrombosis [13]. Actually current guidelines recommend against thromboprophylaxis for patients undergoing knee arthroscopy who do not have a history of VTE [14]. Randomized trial also indicated the use of aspirin in a low-risk population undergoing arthroscopic knee surgery is not warranted, with no cases of postoperative DVT or PE were identified in either arm of the study during the observation period [15]. A multicenter, randomized, controlled trail including 1451 patients regarding prophylaxis with low-molecular-weight heparin for the 8 days after knee arthroscopy reveal it was not effective for the prevention of symptomatic venous thromboembolism, with the number needed to treat (NNT) of 374 [16]. And a randomised study including 241 patients about prophylaxis with Rivaroxaban for the 7 days after knee arthroscopy got the NNT of 8, reach the conclusion whether prophylaxis using Rivaroxaban after surgery should be given to all patients, or to selected “high-risk” subjects, remains to be determined [17].

However, the present study report an unusual case of PE after arthroscopic partial meniscectomy confirmed by ultrasonography and CTA. According to researches identifying the risks of venous thrombosis after arthroscopy of the knee, factors associated with an elevated risk of symptomatic postoperative VTEs included history of malignancy or VTEs, long-term use of anticoagulants, use of estrogen-progestin oral contraceptives or hormone replacement therapy, or history of vascular disease; or (2) two or more of the classic DVT risk factors described in the literature: age older than 65 years, obesity with a body mass index over 30 kg/m2, smoking, oral contraception or hormone replacement therapy, chronic venous insufficiency, and previous DVT [12, 18]. Moreover, a recent study deem these history of “classic” risk factors for venous thromboembolism in patients undergoing routine arthroscopy is very insensitive, preoperative diagnosis of thrombophilia should be both specific and sensitive, including 3 common familial thrombophilias: factor V Leiden, factor VIII, and homocysteine. They were significantly more common in patients who had symptomatic venous thromboembolism after elective routine knee arthroscopy than in healthy normal control subjects, with factor V Leiden heterozygosity (40% vs 2%), high factor VIII level (50% vs 7%), and high homocysteine (30% vs 5%) respectively [19]. Recently, High altitude also is regard as potential risk factor of VTE. The hypoxia result from lower ambient oxygen concentration at higher altitudes can lead to increased platelet aggregation and activation of blood coagulation factors, resulting in a prothrombotic state. A new retrospective case-control study confirmed that isolated arthroscopic partial meniscectomy and/or chondroplasty performed at an altitude ≥4000 ft was a significant risk factor for the development of postoperative VTE compared with matched patients undergoing the same procedure at an altitude less than or equal to 100 ft [20]. After the patient in current case was stable, more information were acquired about the risk factors of VTEs. The common serum tumor markers were detected to screen occult malignancy, and the results were all normal. She had no malignancy,VTEs and vascular diseases history, no use of anticoagulant and hormone. Also she had no classic DVT risk factors but ipsilateral leg varicose veins. And the surgery was performed in Ningbo city, a coastal city in east of China with average altitude of 12.6 ft. No more thrombosis prophylaxis but one dose of LMWH was given after arthroscopic meniscus surgery and discontinued since discharged at following day. She presented the typical clinical manifestation of DVT, swell extended distally from knee joint and developed to pitting edema. It may result from the thrombosis in popliteal vein, which almost obstruct the venous drainage of the distal lower limb. And 1 week later, PE was developed and cause severe symptoms. After the CTA confirmed the etiology, thrombolytic and anticoagulant therapy were given in time and declared her out of danger. The popliteal vein thrombosis and pulmonary embolism vanished eventually. Since the Warfarin was continued after discharge to treat residual small saphenous vein thrombosis, the index of thrombophilias mentioned above haven’t been measured.

An interactive discussion about this topic was published on The New England journal recently, ample reasons were both raised in each arm about receive postoperative thromboprophylaxis or not [1]. Anyway, except the patients with definite risk factors, it is hard to make a clinical decision whether to use preventive anticoagulation after knee arthroscopy according to current guidelines. Routine preoperative measurement of 3 common familial thrombophilias may helpful including factor V Leiden, factor VIII, and homocysteine. In addition, the risk of thrombosis complication should be informed before operation. After surgery, ambulate early should encourage, signs and symptoms of venous thromboembolism need to be educated. By accomplishing these two rules, the incidence rate and hazard of VTEs after arthroscopy may reduce relatively.

## Conclusion

We report a rare case of deep vein thrombosis and pulmonary embolism after arthroscopic meniscus surgery in a 53-year-old woman, who don’t have common risk factors for venous thromboembolism and recover well due to diagnosis and treatment in time. Despite the low incidence after simple arthroscopy surgery, its life-threatening and devastating property make clinicians rethink the necessity of thromboprophylaxis and importance of preoperative relative risk factors screening.

## Abbreviations

CPM: continuous passive motion
CTA: CT angiography
DVT: deep vein thrombosis
FDP: Fibrin degradation product
ICU: Intensive care unit
LMWH: low molecular weight heparin
LPOA: Left popliteal artery
LPOV: Left popliteal vein
NNT: number needed to treat
PE: pulmonary embolism
VTE: venous thromboembolism
